# Duped by dumping syndrome: non-endemic *Vibrio cholerae* bacteremia in an immunocompetent host with gastric bypass surgery, a case report

**DOI:** 10.1099/acmi.0.000517.v3

**Published:** 2023-10-31

**Authors:** Fabiola Reyes, Nicole Pecora, Zoe Freeman Weiss

**Affiliations:** ^1^​ Brigham and Women’s Hospital, Division of Infectious Diseases, 75 Francis St, Boston, MA, 02115, USA; ^2^​ Tufts Medical Center, Department of Pathology, 800 Washington St. Boston, MA, 02111, USA; ^3^​ Brigham and Women’s Hospital, Department of Pathology, 75 Francis St, Boston, MA, 02115, USA; ^4^​ Tufts Medical Center, Division of Geographic Medicine and Infectious Diseases, 800 Washington St. Boston, MA, 02111, USA

**Keywords:** non-epidemic*Vibrio cholerae*, NOVC, bacteremia, gastric bypass, infectious diarrhea, case report

## Abstract

Extra-intestinal infection with non-O1/non-O139 strains of *Vibrio cholerae* (NOVC) is rare, though bacteremia and hepatobiliary manifestations have been reported. Reduced stomach acid, or hypochlorhydria, can increase risk of *V. cholerae* infection. We describe a 42-year-old woman with hypochlorhydria due to untreated *Helicobacter pylori* infection, gastric-bypass surgery, and chronic proton pump inhibitors (PPI) exposure, who developed acute diarrhoea following raw oyster consumption. Her symptoms were attributed to rapid gastric emptying (dumping syndrome) after a negative limited stool work-up. She had persistent diarrhoea, weight loss, and after 5 months was admitted with acute cholecystitis and NOVC bacteremia, requiring cholecystectomy. This is the first reported case of NOVC bacteremia and cholecystitis in a patient with gastric bypass. This case highlights the potential for NOVC biliary carriage, the role of hypochlorhydria as a risk factor for *Vibrio* infection, and the importance of excluding infectious diarrhoea in patients with new onset of symptoms compatible with dumping syndrome and a relevant travel history.

## Data Summary

The datasets generated and/or analysed during the current study are available in the NCBI repository (SAMN27723935).

## Background

Epidemic strains of *Vibrio cholerae *, including the O1 and O139 subtypes, are typically associated with poor drinking water access and suboptimal sanitation. These strains encode the *ctx* (cholera toxin) and *tcpA* (toxin-coregulated pilus) genes which are known to cause severe acute diarrhoea and volume depletion [1]. Over 200 serotypes of *V. cholerae* have been identified. Non-O1/non-O139 strains (NOVC) have been increasingly reported in clinical and environmental settings. *V. cholerae* are found in marine environments including water, sediment, abiotic, and biotic surfaces (e.g. crustaceans exoskeleton) [2]. Growth and survival is optimized in warm, low salinity water. Rising global temperatures and severe precipitation events leading to reduced salinity of ocean waters, have potentiated the global burden of NOVC infection and has led to significant geographic expansion over the last 40 years [3, 4]. Clinical isolates of NOVC have been linked to diarrhoeal illness, wound infection, gastroenteritis, and bacteremia [5–7]. Infection is frequently associated with consumption of seafood (primarily crustaceans and molluscs) [8–10] or soft tissue injuries exposed to seawater [11], primarily in immunocompromised hosts [5, 12].

## Case presentation

**Fig. 1.**
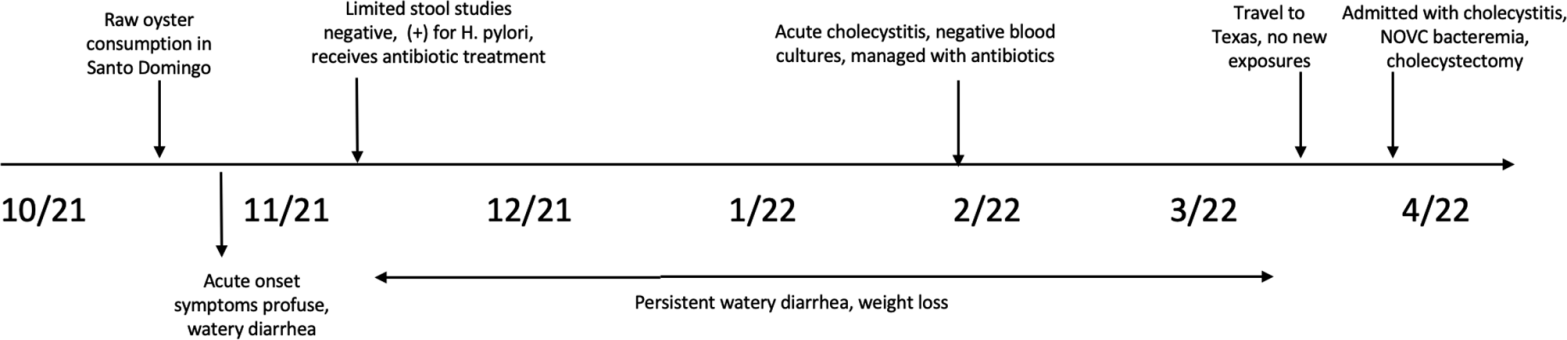
Timeline of events.

A 42-year-old Hispanic female with a history of Roux-en-Y gastric bypass surgery in early 2021 resulting in 110 pound weight loss and incompletely treated *H. pylori* infection (diagnosed in 2018) on chronic omeprazole (>3 years), first presented to a Boston-based clinic in October of 2021 with acute onset diarrhoea and abdominal pain. She had just travelled to Miami, Florida and then Santo Domingo, Dominican Republic, where she reported eating raw oysters (See Fig. 1. Timeline). A few days after her oyster exposure, she developed profuse explosive watery diarrhoea and near syncope. She had a limited initial workup including *Salmonella/Shigella* culture, *Campylobacter* Enzyme Immunoassay (EIA), and Shiga toxin EIA, all of which were negative. *H. pylori* stool antigen was positive. She continued her baseline omeprazole and was treated with amoxicillin and clarithromycin for 2 weeks. Persistent diarrhoea and ongoing weight loss was attributed to dumping syndrome typical of patients with a history of gastric bypass. Subsequent *H. pylori* antigen testing was negative.

In January of 2022, she was evaluated for an episode of acute cholecystitis. Abdominal ultrasound showed a distended gall-bladder with cholelithiasis and positive sonographic murphy sign. No blood cultures or stool studies were sent. She was discharged with a ten-day course of amoxicillin/clavulanic-acid. She continued to have stable watery, loose stools. In March of 2022 she travelled to Texas, but reported no consumption of seafood or water exposures. A few days after her return she again developed worsening abdominal pain, now with vomiting. At this juncture she had lost an additional 30 pounds. Labs were notable for elevated liver enzymes: aspartate aminotransferase 601 IU/L (ref 9–32 IU/L), alanine aminotransferase 349 IU/L (ref 4–37 IU/L), alkaline phosphatase 427 IU/L (ref 40–129 IU/L), total bilirubin 1.2 mg dL^−1^ (ref 0.1 to 1.2 mg dL^−1^), direct bilirubin 0.8 mg dL^−1^ (ref 0.1–0.3 mg dL^−1^). *Clostridium difficile* antigen was negative. Abdominal ultrasound showed cholelithiasis and MRCP was consistent with cholecystitis. Two sets of blood cultures were positive from both aerobic and anaerobic bottles for Gram-negative rods.

**Fig. 2.**
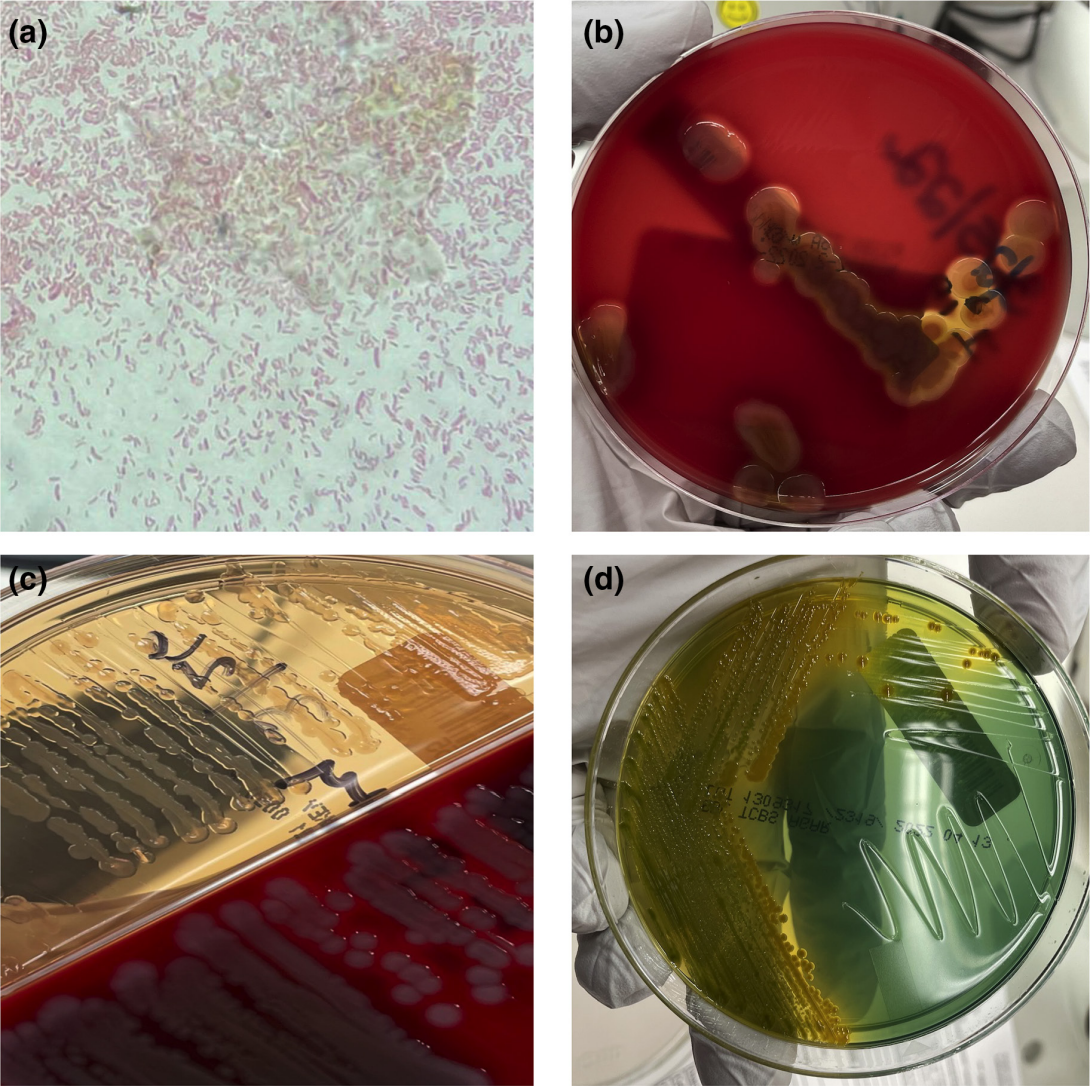
A. Gram-stain of cultured organism demonstrating curved Gram-negative rods. **b.** Colonies on sheep blood agar. Colonies appear to be beta-hemolytic when confluent. Beta hemolysis is less apparent on sheep agar (bottom) when colonies are spread further apart. **c.** Lactose non-fermenting organism demonstrated (top). **d.** Yellow colonies on thiosulphate citrate bile salt sucrose agar (TCBS).

Blood culture Gram-stain demonstrated curved Gram-negative rods (Fig. 2a). Beta hemolysis was observed on sheep blood agar (though the colonies appeared confluent) (Fig. 2b). The isolate was oxidase positive. On MacConkey agar, it appeared non-lactose fermenting (Fig. 2c). It was further subbed to thiosulphate citrate bile salt sucrose agar (TCBS) which demonstrated yellow colonies (Fig. 2c). The isolate was confirmed as *V. cholerae* by MALDI-TOF using the VITEK MS (bioMérieux, France) and then later by the Centres for Disease Control and Prevention (Atlanta, GA) as a member of the nontoxigenic serotype (non-O1, non-O139). The *ctxA* toxigenic marker was negative by multiplex PCR. Stool cultures on antibiotics were negative including on *Vibrio* sp. selective media. The isolate was susceptible to ampicillin, ciprofloxacin, levofloxacin, tetracycline, and trimethoprim-sulfamethoxazole. Her liver function tests improved and she was discharged to complete a 10 day course of ciprofloxacin. Her weight, appetite, and stool consistency improved slowly over the subsequent 2–3 months.

The isolate was submitted for whole genome sequencing (WGS) according to clinical laboratory protocols. Briefly, DNA was extracted using the Qiagen EZ-1 (Qiagen, MD). Libraries were prepared using Illumina DNA Prep (Illumina Inc., CA). WGS was performed with the 600-cycle v3 kit on a MiSeq (Illumina). Raw reads were assembled using SPAdes [13]. Multi-locus sequence type (MLST) was assigned using MLST 2.0 (Centre for Genomic Epidemiology) [14]. Sequences were annotated using Rapid Annotation Subsystem Technology (RAST) [14].

The organism was confirmed to be *V. cholerae* (non-O1/O139), MLST 213 [14] The length of the draft assembled genome was four million base pairs with a N50 of 286 315. No antibiotic resistance genes were identified using ResFinder [15]. It was not associated with any known clusters using the NCBI Pathogen Detection tool (SAMN27723935) [16] or cases of known human infection. Virulence genes detected using the VFDB database tool [17] included those associated with adherence, anti-phagocytosis, chemotaxis, iron uptake, type VI secretion systems, toxin formation (non-ctx-AB or tcpA), biofilm formation, endotoxin, fibrial adherence, and immune evasion (see Table 1).

**Table 1.** Virulence factors detected by whole genome sequencing using the VFDB database [17]. Of note, no antibiotic resistance genes were identified using ResFinder [15]

Virulence factor	Associated genes detected
Adherence	mshH, matD/ecpC, matE/ecpD, hcpB, tapT, tadA, fimC, finD
Anti-phagocytosis	cpsA, mlC, wbjD/wecBM wecC, wza, wzb, wzc, gnd, man b, wbaZ, uge
Chemotaxis	chem flil, plcN, eno
Iron uptake	iucD, entE, ybdA, febB, febG, chuA, chuS, chuU, pvdH
Type VI secretion systems	vasG, aaiA, aaiB, clpV1
Toxin formation	hlyA
Biofilm formation	adeG
Endotoxin production	htrB, kdtA, lpxD, lpxK, msbA, opsX/rfaC, rfAF, wecA
Fibrial adherence	steB, stfD, stiB
Immune evasion	galE, galU

## Discussion and conclusions

NOVC infection is typically acquired by ingestion of infected seafood or wound exposure to marine environments and can result in severe clinical manifestations. Unlike epidemic strains, NOVC isolates typically do not carry the ctxAB and cpA genes. Despite this, NOVC strains are associated with significant genetic diversity and may harbour other virulence factors acquired via horizontal gene transfer. The toxigenic potential of NOVC strains have been attributed to genes coding for type III and type VI secretion systems, enterotoxins, and hemagglutinin proteases [18, 19]. This isolate demonstrated genes coding for a type VI secretion system, associated with bacterial persistence [20], and an hlyA gene, coding for hemolysin, associated with gut colonization and immune evasion, and gastroenteritis in NOVC strains [18, 21].

In addition to pathogen virulence factors, host susceptibility to infection is necessary to result in clinical disease. Reduced stomach acid has been proposed as a possible risk factor for both epidemic and non-epidemic *V. cholerae* infection due to the loss of protection conferred by normal gastric acid levels [22–26]. Our patient was on chronic omeprazole and also had Roux-en-Y gastric bypass surgery which has been associated with decreased basal and peak production of gastric acid [27] She also had a history of untreated *H. pylori* which has been associated with an increased risk of cholera infection [28], possibly due to the induction of hypochlorhydria [29, 30].

NOVC presenting as acute biliary disease has been described in the literature in numerous case reports from both epidemic [31, 32] and non-epidemic strains [33, 34]. In one retrospective review, Chen *et al*. reported that among 83 NOVC infections reported from Taiwan (between 2009 and 2014), 12 patients (14.5 %) presented with biliary tract infection [35]. Biliary carriage of *V. cholerae* has also been suggested, though little has been published describing this phenomenon. In 1967, O1 *V. cholerae* was isolated from duodenal aspiration of two convalescent cholera patients in their post-infection period (7 days or more after their last positive stool culture) after stimulation with cholecystokinin, suggesting the gallbladder as a reservoir [36]. Also in 1967, a patient later named ‘Cholera Dolores’ was identified as a probable long term carrier of *V. cholerae *. She continued to shed morphologically consistent *V. cholerae* in the stool from 1962 to 1966. She had ongoing symptoms of obstructive biliary disease. Duodenal fluid aspiration after a fatty meal clearly indicated the high presence of *V. cholerae* [37]. The persistence of *V. cholerae* in the biliary tract may be due to the production of biofilms in the presence of bile [38], which may form around gallstones. In our patient, subsequent bacteremic events required the formation of biofilms to allow for bacterial persistence over the course of months. Intestinal and biliary biofilm formation by *V. cholerae* is an important precursor to systemic disease [2 ].

Treatment of invasive NOVC infection typically requires systemic antibiotics (e.g. ampicillin, piperacillin-tazobactam, ceftriaxone, cefepime, ciprofloxacin, or carbapenems). Drug resistance in NOVC strains has been rising steadily, with high rates of ampicillin resistance (up to 88 % in some settings) and, though rare, increasing reports of ciprofloxacin resistance word-wide (from 3 % by 2010–7 % in 2020) [39]. Susceptibility on extra-intestinal isolates should be performed, as drug resistance will impact antibiotic selection.

To our knowledge this is the first reported case of NOVC cholecystitis and bacteremia in a patient with prior gastric bypass surgery. Though not immunocompromised, this patient’s primary risk factor for systemic infection was her recent gastric bypass procedure and the resultant hypochlorhydria. *H. pylori* infection and PPI use may have also played a role. Her most likely exposure (oyster consumption) and compatible clinical history was 5 months prior to developing bacteremia, though we cannot exclude the possibility of a more recent exposure. While her persistent diarrhoea may have been related to dumping syndrome, any superimposed infectious diarrhoea was missed due to a limited stool workup. *V. cholerae* would not have been detected from the stool workup performed and further stool studies were not pursued until after the diagnosis of *V. cholerae* was made. She did receive antibiotic therapy for *H. pylori* and the first episode of cholecystitis that were active against *V. cholerae *. It is possible that she entered into a carrier state following her initial infection, and/or that antibiotics were poorly absorbed due to malabsorption. In other biliary infections, such as with *Salmonella typhi,* biliary carriage is not easily eradicated with antibiotics due to biofilm formation on gallstones [40].

This case highlights the need for appropriate microbiological testing based on patients’ exposure history and the clinical importance of excluding infectious diarrhoea in patients with new onset of symptoms or ongoing symptoms compatible with dumping syndrome and a relevant travel history. Clinical microbiology labs may offer *Vibrio* testing through expanded gastrointestinal multiplex PCR panels or through clinician-directed requests for *Vibrio* cultures. Clinicians should be aware of their institutional protocols for stool culture workup, as *Vibrio* is often not routinely assessed. We highlight gastric bypass surgery as a risk factor for *V. cholerae* infection. Further studies are needed to understand the role of gallbladder carriage in *V. cholerae *.
